# Comment on “Soluble Urokinase-Type Plasminogen Activator Receptor Plasma Concentration May Predict Susceptibility to High Altitude Pulmonary Edema”

**DOI:** 10.1155/2017/8546027

**Published:** 2017-02-08

**Authors:** Gaurav Sikri, Srinivasa Bhattachar

**Affiliations:** Department of Physiology, Armed Forces Medical College, Solapur Road, Wanowrie, Pune 411 040, India

We read with profound interest the article titled “Soluble Urokinase-Type Plasminogen Activator Receptor Plasma Concentration May Predict Susceptibility to High Altitude Pulmonary Edema” by Hilty et al. [1]. The authors have concluded that soluble urokinase-type plasminogen activator receptor (suPAR) plasma concentration measured before hypoxic exposure may predict susceptibility of a lowlander to high altitude pulmonary edema (HAPE). Despite the fact that inflammation is associated with acute hypoxia exposure in both HAPE and acute mountain sickness (AMS), studying these two high altitude illnesses together in the same cohort in this study seems to be convoluted. AMS and HAPE are known to have pathophysiological processes involving central nervous system and cardiopulmonary systems, respectively, with hypoxia being the common triggering factor [2]. This has also been indirectly acknowledged by the authors, while suggesting that cellular based inflammation does not play a role in the central form of high altitude disease, comprising AMS and high altitude cerebral edema. Moreover, occurrence of HAPE may not always be associated with AMS [3]. Though the role of inflammation in priming pulmonary endothelium towards hypoxia-related pulmonary edema is well established, the study of the biomarker with respect to AMS is intriguing.

Acute hypoxia exposure is associated with sympathetic activation, which results in rise in heart rate (HR) [4]. However, on the contrary as per Table 1 of the study, in dexamethasone prophylaxis group of HAPE susceptible subjects (*n* = 10), HR was found to decrease from their sea level HR of 74/min to 68/min after hypoxia exposure. As physiologists with experience of working in high altitude physiology, we contemplate if use of dexamethasone in these individuals resulted in these changes.

HAPE is a disease known to occur two or more days after exposure to altitudes above 3000 m [5]. However, subjects in this study were assessed for HAPE after 24 hours of hypoxia exposure at Margherita Hut (4559 m), and an insignificant difference was seen in suPAR levels of HAPE susceptible and nonsusceptible individuals. Subjects were given dexamethasone 24 hours after hypoxia exposure as part of another research project. Otherwise serial measurements of suPAR in clinical overt HAPE subjects, if any, occurring over next 4 days of observation could have helped in establishing suPAR as a possible biomarker for HAPE susceptibility. The outcome of the bigger research project, of which the present work is a part, will definitely be worth a wait, which might mark the end of the pursuit of a well-established biomarker for a potentially fatal but preventable disease like HAPE.
